# Switching Algorithm for Maglev Train Double-Modular Redundant Positioning Sensors

**DOI:** 10.3390/s120811294

**Published:** 2012-08-15

**Authors:** Ning He, Zhiqiang Long, Song Xue

**Affiliations:** College of Mechatronics Engineering and Automation, National University of Defense Technology, Changsha 410073, China; E-Mails: hening0606@126.com (N.H.); songself@126.com (S.X.)

**Keywords:** maglev train, positioning sensor, switching algorithm, wavelet analysis, adaptive prediction

## Abstract

High-resolution positioning for maglev trains is implemented by detecting the tooth-slot structure of the long stator installed along the rail, but there are large joint gaps between long stator sections. When a positioning sensor is below a large joint gap, its positioning signal is invalidated, thus double-modular redundant positioning sensors are introduced into the system. This paper studies switching algorithms for these redundant positioning sensors. At first, adaptive prediction is applied to the sensor signals. The prediction errors are used to trigger sensor switching. In order to enhance the reliability of the switching algorithm, wavelet analysis is introduced to suppress measuring disturbances without weakening the signal characteristics reflecting the stator joint gap based on the correlation between the wavelet coefficients of adjacent scales. The time delay characteristics of the method are analyzed to guide the algorithm simplification. Finally, the effectiveness of the simplified switching algorithm is verified through experiments.

## 1. Introduction

**Figure 1.**
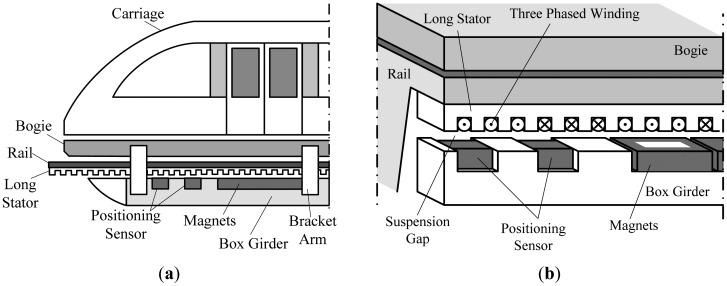
(**a**) Sketch map of a high speed maglev train; (**b**) Sketch map of the substructure of a high speed maglev train.

The suspension function of high speed maglev trains is carried out by the electromagnetic attractive force between the electromagnets and the rail, and the train is driven by a linear synchronous motor [1–3]. The 3-phased primary windings are inlaid in the slots of the long stator fixed along the rail, and the secondary windings are the electromagnets shown in Figure 1. In order to implement high efficient synchronous traction, the traction system needs the precise relative position between the electromagnets and the long stator.

Because of the non-contact between the train and the rail, the technical requirements for the position and speed measurements of a maglev train are different from those for wheel-track systems [4]. Considering the dimensional accuracy of the tooth-slot structure of the long stator, high precision positioning with millimeter-sized resolution can be achieved by detecting the tooth-slot structure through variable magnetic-resistance type sensors [5–9].

Detecting coils fixed on one side of the positioning sensor facing the long stator are driven by high frequency signal sources. Taking one of the coils for example, when the coil moves along the stator at a certain suspension gap, its inductance varies periodically. As a result, the amplitude of the coil signal varies accordingly forming an amplitude modulation signal. Then, a square signal corresponding to the tooth-slot structure can be obtained by comparing the amplitude demodulation signal to a certain threshold.

By counting the jumping edges of the square signal, the number of tooth-slot periods passed by the train can be determined and the positioning with higher resolution in a tooth-slot period can be achieved by looking up the mapping table between the sampled values of the amplitude demodulated signals and the relative position.

**Figure 2.**
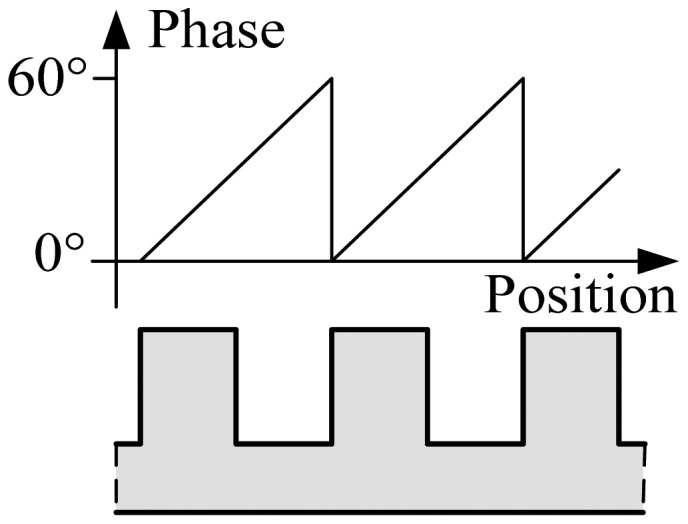
The relationship between the electrical phase angle and the tooth-slot structure.

A magnetic pole period of the 3-phased windings contains six tooth-slot periods as shown in Figure 1(b). Thus, the positioning in a tooth-slot period can be denoted by an electrical phase angle between 0° and 60°, as shown in Figure 2.

**Figure 3.**
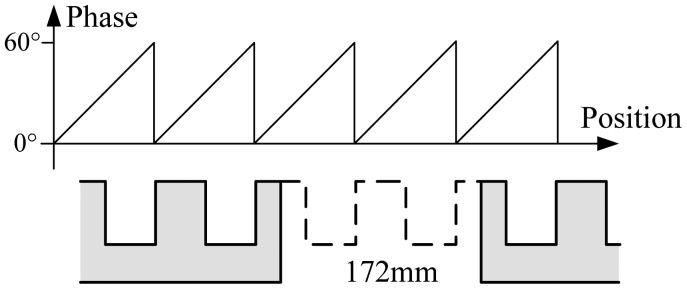
The phase requirement at a large joint gap.

In practice, due to installation requirements, there are some large joint gaps between long stator sections. The length of a gap is about 2 tooth-slot periods as shown in Figure 3. When the sensor is moving below a large joint gap, the positioning signals are invalidated, but the traction system still requires normal positioning signals as shown in Figure 3, so redundant positioning sensors are needed. Because of the space constraints of the train structure, only two-modular redundancy is adopted, which increases the difficulty in the switching algorithms.

In order to identify the invalidated positioning signal in time, fault diagnosis technologies can be adopted. Generally speaking, fault diagnosis technologies can be classified into three categories: methods based on system models, methods based on signal processing and methods based on knowledge. Because model parameters such as carriage mass, tractive force, electrical brush friction and slop grade of the rail are unknown to the positioning sensor, methods based on model are not feasible. Besides, methods based on knowledge usually require complicated inference procedures and knowledge bases, so it's hard for these methods to satisfy the time limit in this situation. Therefore, the methods based on signal processing are considered to implement the switching of the positioning sensors in real time.

In [8] some simulation studies of the two-modular switching algorithms for the positioning sensor based on adaptive filter are performed. However, the waveform of the signals collected in actual runs of a maglev train is much worse than that of the simulated signals because of various disturbances and uncertainties in practical situation. Therefore, the performances of the method mentioned in [8] will be reduced considerably in practical application.

In order to enhance the reliability of the switching algorithm, wavelet analysis is adopted to suppress measuring disturbances without weakening the signal characteristics caused by the stator joint gaps based on the correlation between the wavelet coefficients of adjacent scales in this paper. The time delay characteristics of the method are analyzed to guide the algorithm simplification. Finally, the effectiveness of the simplified algorithm is proven through simulations and experiments.

## 2. Analysis of Positioning Signals near Large Joint Gaps

**Figure 4.**
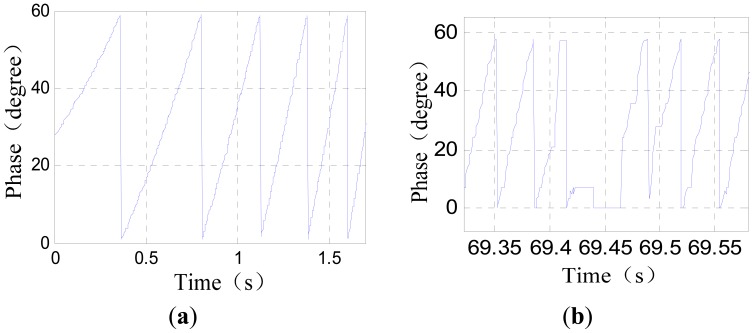
(**a**) Normal phase signal; (**b**) phase signal near a large joint gap.

Figure 4 shows the phase signal waveforms of a positioning sensor recorded during a test run.

As Figure 4 shows, the phase signal near a large joint gap has serious waveform distortions. A large joint gap will also causes tooth-slot period number counting losses, that is to say, the tooth-slot period number obtained by the sensor will be one less than the number required by the traction system. When the sensor has passed across a large joint gap, the phase signal will become normal again, but the phase error caused by the tooth-slot period number counting loss will be accumulated and cannot be corrected automatically. The counting loss of one tooth-slot period corresponds to a phase lag of 60°. The phase difference will break the synchronization between the traveling magnetic field and the electromagnets' magnetic field, reduce the efficiency of the traction considerably and even cause overcurrent protection or damages to the traction system. If the accumulated phase difference reaches 180° after passing several these gaps, the traction system will generate a wrong tractive force with an unexpected direction. This is a potential safety hazard. Therefore, the switching algorithm should identify the signal distortion caused by the large gaps accurately in time, and switch to the other positioning sensor before counting loss happens, and then correct the tooth-slot period count value of the invalid senor according to that of the normal one.

The jumping edges of the sawtooth phase wave containing lots of high frequency harmonic components will complicate the signal prediction and characteristics extraction. Therefore, firstly the phase signal needs to be converted to a certain form to eliminate the influence of the jumping edges. Let *p_h_*(*k*) denote the current phase value and *n*(*k*) denote the current tooth-slot period number. Considering a tooth-slot period corresponds to a phase angle of 60°, the converted signal is calculated as follows [10]:

(1) $$p_{ha}(k)=60n(k)+p_{h}(k)$$

Because of the slight time differences between the jumping edges of the phase signal and those of the tooth-slot number signal, there are spike pulses in signal *p_ha_*(*k*). The spike pulses can be eliminated through simple logical pretreatment [10]. As the spike pulses are about 60°, so they can be identified by detecting the difference value of the adjacent sampling phases. If the phase difference is about 60°, and then make the current sampling phase equal to the former, so that the spike pulses can be eliminated.

**Figure 5.**
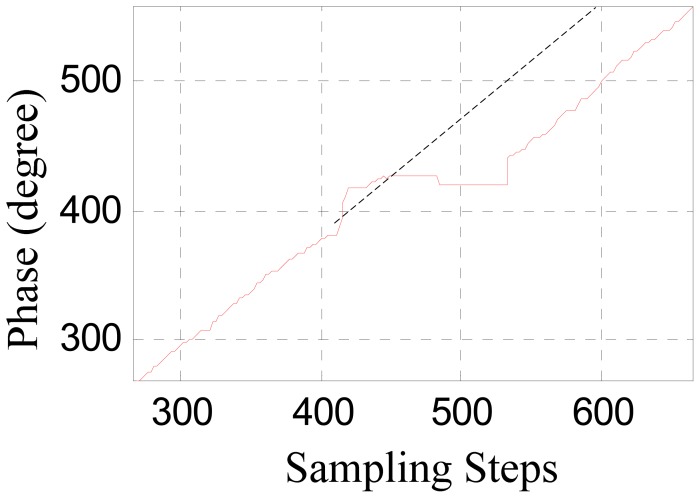
The converted phase signal.

The converted phase signal near a large joint gap is shown in Figure 5, where the dashed line denotes the converted signal of an ideal phase signal. It can be seen that the tooth-slot period number counting loss results in a phase lag.

## 3. Switching Algorithm Based on Adaptive Linear Prediction

The converted phase signal is not a stationary random process. Its statistical properties vary continually. In this case, the least-mean-square-error adaptive linear prediction is applicable to predict the phase signal based on the evaluation of the short time statistics of the signal.

The basic idea of the switching algorithm is to predict *p_ha_*(*k*) according to historical data: *p_ha_*(*k* – 1), *p_ha_*(*k* − 2), …, *p_ha_*(*k* − *m*). Then, prediction error can be obtained as *e*(*k*) = *p_ha_*(*k*) − *p̂_ha_*(*k*), where *p̂_ha_*(*k*) denotes the predicted value of *p_ha_*(*k*). Thus the sensor switching can be triggered by comparing the prediction error to a suitable threshold.

Let *m* denote the length of the predictive filter, and the predicted value *p̂_ha_*(*k*) can be calculated as follows [11]:

(2) $${\hat{p}}_{ha}(k)=\text{w}{\left(k\right)}^{\text{T}}p_{ha}(k)$$

where:

(3) $${\text{p}}_{ha}(k)=\left[\begin{array}{cccc} p_{ha}(k-1)] & p_{ha}(k-2) & \cdots & p_{ha}{\left(k-m\right)}^{\text{T}} \end{array}\right]$$

(4) $$\text{w}(k)={\left[\begin{array}{cccc} w_{1}(k) & w_{2}(k) & \ldots & w_{m}(k)] \end{array}\right]}^{\text{T}}$$

and ***w***(*k*) is the weight vector of the predictive filter. The adaptive process of the weight vector based on the least-mean-square-error criterion is given as follows:

(5) $$\text{w}(k+1)=\text{w}(k)+\mu {\text{p}}_{ha}(k)e(k)$$

Reference [11] gives the range of *μ* to make the iterative process of Equation (5) converge when *m* is relatively big, but the phase signal varies quickly when the train is running, so the nonstationarity degree of the signal is high. Therefore, *m* should be set to a small value. In this case, the upper limit for *μ* can't be exactly calculated. So, in this paper, *μ* is given experientially as follows:

(6) $$\mu =1/\lambda_{\max}$$

where *λ*_max_ denotes the maximal eigenvalue of the matrix ***p****_ha_*(*k*)***p****_ha_*(*k*)^T^.

**Figure 6.**
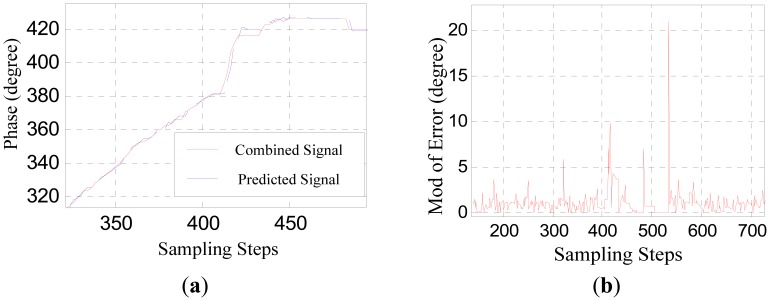
(**a**) The predicted phase signal; (**b**) The prediction error.

Figure 6(a) shows the contrast between the converted phase signal and the predicted signal obtained through the adaptive linear prediction method discussed above. Figure 6(b) shows the absolute value of the prediction error.

In Figure 6(a), the jumping distortion of the waveform at about the 410th step indicates that the sensor is beginning to be affected by the large joint gap. This jumping distortion corresponds to the peak at about the 410th step in Figure 6(b). The peak at about the 534th step indicates that the sensor is moving out of the influence range of the large joint gap.

However, as Figure 6(b) shows, the prediction error peaks due to the large joint gap is not larger than the prediction error due to measurement noise. Considering there are various uncertainties during practical runs of a maglev train, sensor-switching based on straightforwardly comparing the prediction error to a certain threshold may result in false operation or switching failure. Therefore, it is necessary to further amplify the difference between the signal characteristics reflecting large joint gaps and that of the measurement disturbances.

## 4. Noise Suppression Pretreatment Based on Wavelet Analysis

Low Pass Filters (LPF) can be used to smooth the converted signal and suppress noise, but they will also weaken the signal characteristics reflecting the stator joint gaps at the same time, whereas, the method based on the correlation between the wavelet coefficients of adjacent scales [12] can suppress measuring disturbances without weakening the signal characteristics.

Generally, for a signal, the Lipschitz index is larger than zero at continuous sections and equal to zero at step type discontinuous points, whereas, the Lipschitz index of a noise signal is less than zero. Accordingly, the wavelet coefficients of the three cases have different propagation characteristics on each transformation scales. For the former two cases, the wavelet coefficients of adjacent scales have a relatively strong correlation, but for noise signal, the correlation is not obvious. Hence, by multiplying each wavelet coefficient of a scale by the corresponding coefficient of an adjacent scale respectively, the noise can be suppressed, and at the same time, the valid signal characteristics are enhanced [12].

For a discrete parameter wavelet transformation, the numbers of the wavelet coefficients of different scales are not the same because of binary down sampling, so it's not feasible to do the one-to-one multiplication for the coefficients of adjacent scales straightforwardly. In order to solve this problem, stationary wavelet transform algorithm (a'trous algorithm) is adopted to make the number of the wavelet coefficients of each scale equal to the length of the original data when finite length problem is not considered.

Consider an orthogonal discrete parameter wavelet with a limited support interval. Let ***h*** denote the low pass analytical filter determined by the wavelet and ***g*** denote the corresponding high pass analytical filter. So ***h*** and ***g*** are Finite Impulse Response (FIR) filters. Suppose the length of the filter is 2*N*. Let ***h****^j^* and ***g****^j^* respectively denote the filters of the *j* th scale obtained by inserting (2*^j^* − 1) zero elements behind each element of ***h*** and ***g***. They are expressed as follows:

(7) $${\text{h}}^{j}=[h_{0}^{j}h_{1}^{j}\cdots h_{2^{j+1}N-1}^{j}]$$

(8) $${\text{g}}^{j}=[g_{0}^{j}g_{1}^{j}\cdots g_{2^{j+1}N-1}^{j}]$$

Let *p_ha_^j^*(*k*) and *d^j^*(*k*) respectively denote the scale coefficients and the wavelet coefficients obtained by applying stationary wavelet transform to the signal *p_ha_^j^*(*k*) on the *j* th scale. They are calculated as follows:

(9) $${p_{\text{h}a}}^{j+1}(k)=\left[{p_{ha}}^{j}(k){p_{ha}}^{j}(k+1)\cdots {p_{ha}}^{j}(k+2^{j+1}N-1)\right]h^{j\text{T}}$$

(10) $$d^{j+1}(k)=\left[{p_{ha}}^{j}(k){p_{ha}}^{j}(k+1)\cdots {p_{ha}}^{j}(k+2^{j+1}N-1)\right]{\text{g}}^{j\text{T}}$$

*p_ha_^j^*(*k*) can be perfectly reconstructed as follows:

(11) $${p_{\text{h}a}}^{j+1}(k)=\left[{p_{ha}}^{j+1}(k){p_{ha}}^{j+1}(k-1)\cdots {p_{ha}}^{j+1}(k-2^{j+1}N+1)\right]h^{j\text{T}}+\left[d^{j+1}(k)d^{j+1}(k-1)\cdots d^{j+1}(k-2^{j+1}N+1)\right]{\text{g}}^{j\text{T}}$$

According to Equations (9,10), *d^j^*(*k*) is the linear combination of *p_ha_*(*k*), *p_ha_*(*k* + 1), …, *p_ha_*(*k* + 2*^j^*^+1^*N* − 2*N* − *j*), and *d^j^*^+1^(*k*) is the linear combination of *p_ha_*(*k*), *p_ha_*(*k* + 1), …, *p_ha_*(*k* + 2*^j^*^+2^*N* − 2*N* − *j* − 1). So *d^j^*(*k*), …, *d^j^*(*k* + 2^j+1^*N* − 1) all have strong correlations with *d^j^*(*k*) respectively. In order to enhance the signal characteristics and suppress noise more effectively, it's necessary to find a certain *d^j^*(*k*′) which has the maximal correlation with *d^j^*^+1^(*k*), and then multiply *d^j^*(*k*′) by *d^j^*^+1^(*k*). Here, an empirical formula to choose *k*′ is given as follows:

(12) $$k\prime =k+\mathrm{int}(c(2^{j+1}N-1)),c\in [0,1]$$

For different wavelets, *c* is different and can be determined by experiments.

**Figure 7.**
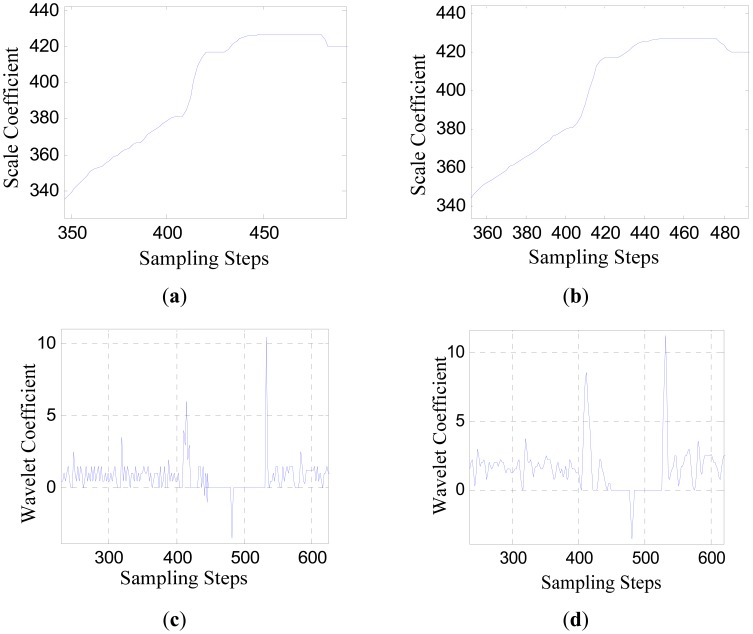
(**a**) The scale coefficients on the scale *j* = 1; (**b**) The scale coefficients on the scale *j* = 2; (**c**) The wavelet coefficients on the scale *j* = 1; (**d**) The wavelet coefficients on the scale *j* = 2.

The analytical results of the converted signal *p_ha_*(*k*) based on stationary wavelet transform algorithm are shown in Figure 7. The signal is transformed on two scales (*j* = 1,2). Figure 7(a,b) show the scale coefficients, and Figure 7(c,d) show the wavelet coefficients.

Considering the time limit and calculation load requirement of the sensor's practical operating condition, the “db1” wavelet (“haar” wavelet) is selected, which has the shortest filter length with *N* = 1, and is not affected by the finite length problem.

**Figure 8.**
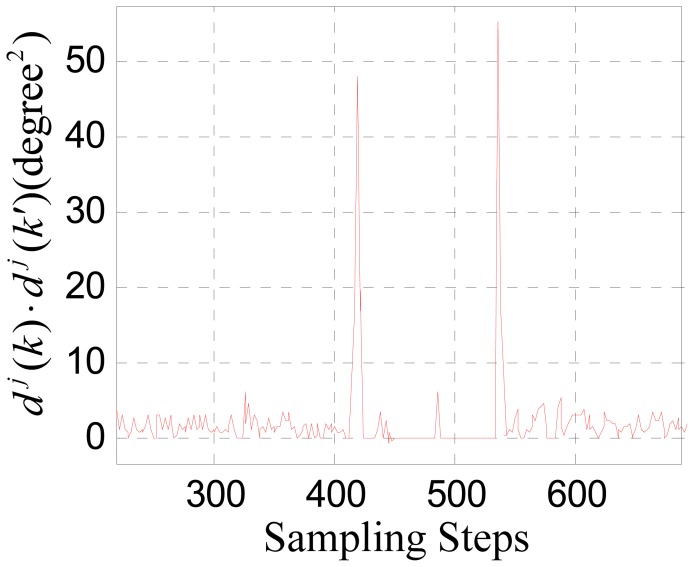
The product of the wavelet coefficients of the 1st and 2nd scales.

Figure 8 shows the waveform of *d^j^*(*k*) &middot; *d^j^*(*k*′), where *c* = 0.5. It can be seen that after the multiplication between the wavelet coefficients of adjacent scales, the wavelet coefficients indicating the moments when the sensor is moving into and out of the influence range of the large joint gap are enhanced obviously.

**Figure 9.**
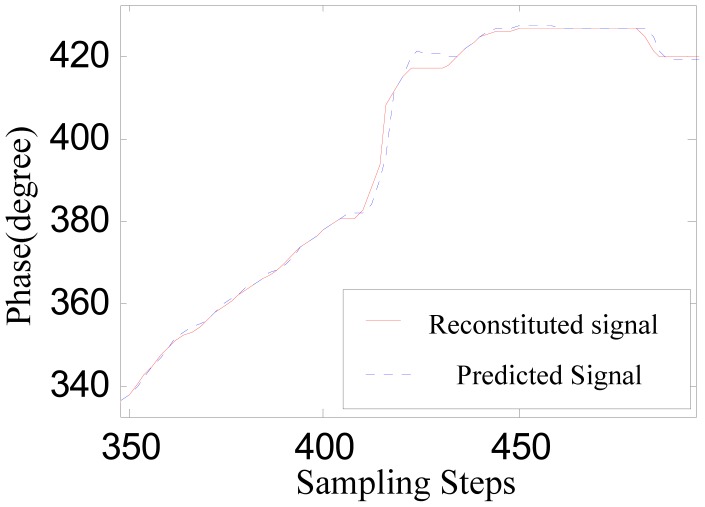
The reconstituted signal and predicted signal.

Choose a threshold *T* = 10, and update the wavelet coefficients by setting *d^j^*(*k*) and *d^j^*(*k*′) to zero when *d^j^*(*k*) &middot; *d^j^*(*k*′) < *T*. Then a reconstructed signal can be gotten by applying Equation (11) to the scale coefficients and the updated wavelet coefficients shown in Figure 9.

Compared to the original signal *p_ha_*(*k*), the reconstructed signal is smoother without weakening some key characteristics. The predicted signal according to the reconstructed signal based on adaptive linear prediction is also shown in Figure 9.

**Figure 10.**
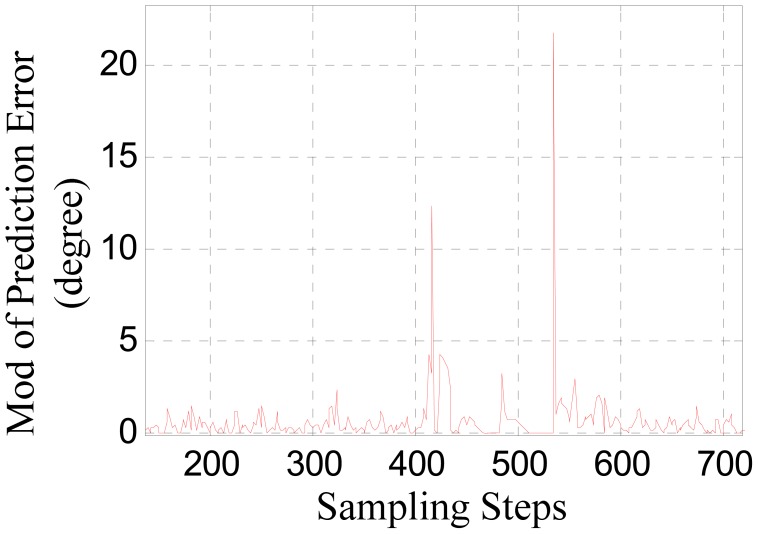
The prediction error.

Figure 10 shows the prediction error. In contrast with Figure 6(b), after the noise suppression pretreatment, the characteristic distinctions between the signal distortions caused by large joint gaps and the measurement noise are amplified effectively. So the effectiveness and reliability of the sensor switching decision can be improved by comparing the signal shown in Figure 10 to a suitable threshold.

## 5. Time Delay Characteristics Analysis of the Switching Algorithm

At first, we consider the switching method based on adaptive linear prediction without the noise suppression process. Because the prediction error *e*(*k*) can be figured out at the same sampling period when *p_ha_*(*k*) is obtained, the time delay of this method is within a sampling period.

When the discrete wavelet transformation is introduced into the process, the finite length problem (boundary effect) needs to be considered except for the “db1” wavelet. The data affected by the boundary effect are always the latest sampled values of the phase signal. Considering the algorithm based on Equations (9,10), boundary effect means that the length of the transformation coefficients is shorter than the original signal, and the serial number of the latest transformation coefficient is smaller than that of the latest sampled signal. This means the signal characteristics reflected by the latest coefficient is lag behind the latest sampled signal. As a result, the switching moment will be postponed for several sampling steps. Usually, the time delay of the switching will be more serious, if the length of FIR wavelet filters and the number of the transformation layers (scales) increase.

One way to solve the boundary problem is to add certain data behind the latest sampled datum to extend the original signal artificially until the serial number of the latest transformation coefficient is equal to that of the latest sampled value. However, the added data are different from the real data sampled later, so the corresponding coefficients can't reflect the signal characteristics exactly.

References [13,14] investigate the boundary effect of the discrete wavelet transform. The Gram-Schmidt orthogonalization method is adopted to orthogonalize the boundary vectors of the wavelet transformation matrix. This technique can guarantee the orthogonality and reversibility of the transformation for a finite-length data sequences, however, the orthogonalized boundary vectors lose the frequency-selective function. The wavelet coefficients and scale coefficients corresponding to these orthogonalized vectors still cannot reflect the detailed information and rough tendency of the original signals clearly. Thus these coefficients are actually invalid data, and can't be used for switching decision. Therefore, the switching delay caused by the inherent boundary problem of wavelet transformation can't be solved nicely except for choosing a wavelet filter with a small *N*.

Furthermore, the signal reconstruction will also introduce a delay. Let *p_ha_*(*k*) denote the datum at the jumping distortion point indicating the sensor is beginning to be affected by the large joint gap. Let *j* denote the scale of the wavelet transformation, and the scale of the original data is denoted as *j* = 0. Denote the latest wavelet coefficient and scale coefficient on the *j*th scale obtained based on Equations (9,10) according to the data *p_ha_*(*k*), *p_ha_*(*k* − 1), *p_ha_*(*k* − 2), … by *d^j^*(*k_j_*) and *p_ha_^j^*(*k_j_*). The serial number *k_j_* can be calculated as follows:

(13) $$\begin{array}{cc} k_{j} & =k-(2^{1}N-1)-(2^{2}N-1)-\cdots -(2^{j}N-1)\\ & =k-2^{j+1}N+2N+j \end{array}$$

Furthermore, we denote the serial number of the latest reconstructed datum on the 0^th^ scale obtained based on Equation (11) according to the wavelet coefficients and scale coefficients on the *j*th scale with serial numbers no bigger than *k_j_* by *k_0_. k_0_* can be gotten according to Equation (11) as follows:

(14) $$k_{0}=k_{j}=k-2^{j+1}N+2N+j$$

The analysis above indicates that the switching algorithm combining the adaptive linear prediction and the stationary wavelet transformation has a time delay of about 2*^j^*^+1^*N*−2*N*−*j* steps. The sampling period of the positioning sensor is 500 μs. Considering the “db1” wavelet is chosen in Section 4 and the sampled signal is transformed to the 2nd scale, the time delay will be about 2 ms.

In engineering practice, the positioning sensor is only enabled when the train is running at a speed below 20 km/h. When the running speed of the train exceeds 20 km/h, the position and phase information can be obtained by detecting the back electromotive force of the primary windings. In a time span of 2 ms, the train can run a distance of about 11 mm with a speed of 20 km/h. Considering that the length of a tooth-slot period is about 86 mm, this algorithm can avoid the tooth-slot period number counting loss in time.

In order to further reduce the time delay and computation needs, the algorithm discussed in Section 4 needs to be simplified. Actually, after the noise suppression pretreatment, the signal characteristics due to the stator joint gap has already been distinguished from the noise obviously according Figure 8. So the switching can be triggered directly by comparing the product of the wavelet coefficients of adjacent scales to a suitable threshold. Thus, the reconstruction and the adaptive prediction processes can be truncated to simplify the algorithm considerably.

According to Equation (12), the serial number of the original signal which is mostly relevant to the *k_j_*th wavelet coefficients on *j* th scale can be calculated as follows:

(15) $$\begin{array}{cc} k_{0} & =k_{j}+\mathrm{int}(c(2^{j}N-1)+c(2^{j-1}N-1)+\cdots +c(2^{1}N-1))\\ & \approx k-\mathrm{int}((1-c)((2^{j}N-1)+(2^{j-1}N-1)+\cdots +(2^{1}N-1)))\\ & =K-\mathrm{int}((1-c)(2^{j+1}N-2N-j)) \end{array}$$

That is to say, if *p_ha_*(*k*) is the datum at the first jumping distortion point, the corresponding peak of the product signal of the wavelet coefficients will be postponed for about int((1 − *c*)(2*^j^*^+1^*N* − 2*N* − *j*)) steps. Comparing Equation (15) with Equation (14), it can be seen that after the algorithm simplification, the time delay reduced by about int(*c*(2*^j^*^+1^*N* − 2*N* − *j*) steps. When *N* = 1, *j* = 2, *c* = 0.5, and the sampling period is still 500 μs, the time delay is about 1ms. In addition, with the simplified algorithm, a shorter sampling period can be adopted to further decrease the switching delay.

## 6. Switching Experiments of the Sensor

The experiments are carried out on the 1.5 km high speed maglev train test line in Shanghai, China. Large joint gaps shown in Figure 3 are located on the test line at intervals. The two-modular redundant positioning sensors are installed on the box girder of the maglev train as shown in Figure 1 with a distance about three tooth-slot periods (258 mm), so the phase signals of the two sensors are almost the same, and even though with the switching between the phase signals, the final phase signal is still continuous.

**Figure 11.**
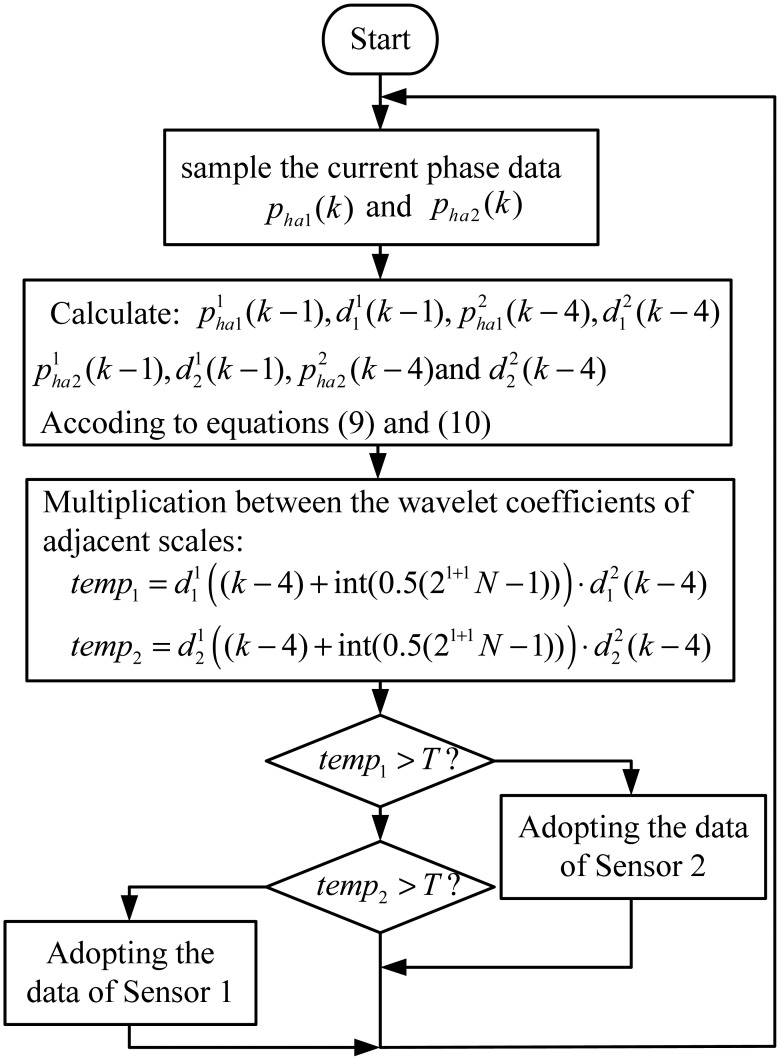
The flow chart of the switching algorithm.

The sensors are connected with an upper computer via communication cables, and upload the phase signal via RS485 interface in real time. The upper computer identifies the signal characteristics due to the joint gaps based on the correlation between the wavelet coefficients of adjacent scales and then implement the sensor switching. The flow chart of the switching algorithm of the upper computer is shown in Figure 11.

**Figure 12.**
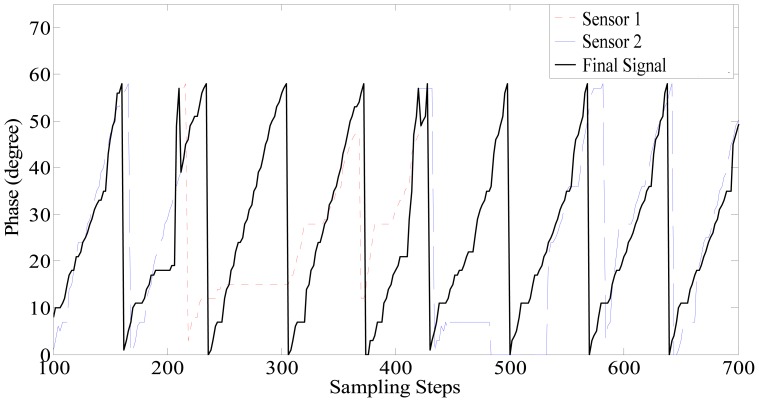
The experimental results of the two-modular switching.

The switching experiment results are shown in Figure 12. For a maglev train, there are two positioning sensors in one set of speed and position detection system. As Figure 11 shows, when the train is passing a large joint gap, the phase signal distortions of two sensors occur one after another. At about the 220th step, the phase signal of sensor 1 (denoted by the red dash line) begins to be distorted, and then the final signal (denoted by the black line) is switched to sensor 2, which is normal at present. About three tooth-slot periods later (at about the 440th step), the phase signal of sensor 2 (denoted by the blue line) begins to be distorted, and then the final signal is switched to sensor 1. Therefore, when the train is passing a large joint gap, the final signal always keeps normal.

From Figure 11, it can be seen that the switching algorithm can effectively avoid the accumulated phase errors caused by the tooth-slot period number counting loss. However, because of the switching delay, the waveform distortions near the switching moments are not eliminated completely. This situation can be improved by introduce proper filtering and shaping processes [10] into the algorithm.

## 7. Conclusions

This paper studies the two-modular switching algorithms for the positioning sensors to solve the problem caused by the stator joint gaps. At first, adaptive filtering is applied to predict the phase signal of the sensor, and the switching is triggered based on the prediction error. In order to enhance the reliability and effectiveness of the switching algorithm, wavelet analysis is introduced in to suppress measuring disturbance without weakening the signal characteristics affected by the stator joint gaps based on the correlation between the wavelet coefficients of adjacent scales. To improve the response speed of the algorithm, a simplified algorithm is proposed, and its time delay characteristic is analyzed. The analytical and the experimental results show that when the train is running at a speed below 20 km/h, the designed algorithm can switch the positioning sensors in good time and can effectively eliminate the accumulated phase errors due to the tooth-slot period number counting losses.
